# Expression of Versican 3′-Untranslated Region Modulates Endogenous MicroRNA Functions

**DOI:** 10.1371/journal.pone.0013599

**Published:** 2010-10-25

**Authors:** Daniel Y. Lee, Zina Jeyapalan, Ling Fang, Jennifer Yang, Yaou Zhang, Albert Y. Yee, Minhui Li, William W. Du, Tatiana Shatseva, Burton B. Yang

**Affiliations:** 1 Sunnybrook Research Institute, Sunnybrook Health Sciences Centre, Toronto, Ontario, Canada; 2 Life Science Division, Graduate School at Shenzhen, Tsinghua University, Shenzhen, Guangdong Province, China; 3 Department of Laboratory Medicine and Pathobiology, University of Toronto, Toronto, Ontario, Canada; City of Hope National Medical Center, United States of America

## Abstract

**Background:**

Mature microRNAs (miRNAs) are single-stranded RNAs that regulate post-transcriptional gene expression. In our previous study, we have shown that versican 3′UTR, a fragment of non-coding transcript, has the ability to antagonize miR-199a-3p function thereby regulating expression of the matrix proteins versican and fibronectin, and thus resulting in enhanced cell-cell adhesion and organ adhesion. However, the impact of this non-coding fragment on tumorigenesis is yet to be determined.

**Methods and Findings:**

Using computational prediction confirmed with *in vitro* and *in vivo* experiments, we report that the expression of versican 3′UTR not only antagonizes miR-199a-3p but can also lower its steady state expression. We found that expression of versican 3′UTR in a mouse breast carcinoma cell line, 4T1, decreased miR-199a-3p levels. The decrease in miRNA activity consequently translated into differences in tumor growth. Computational analysis indicated that both miR-199a-3p and miR-144 targeted a cell cycle regulator, Rb1. In addition, miR-144 and miR-136, which have also been shown to interact with versican 3′UTR, was found to target PTEN. Expression of Rb1 and PTEN were up-regulated synergistically in vitro and in vivo, suggesting that the 3′UTR binds and modulates miRNA activities, freeing Rb1 and PTEN mRNAs for translation. In tumor formation assays, cells transfected with the 3′UTR formed smaller tumors compared with cells transfected with a control vector.

**Conclusion:**

Our results demonstrated that a 3′UTR fragment can be used to modulate miRNA functions. Our study also suggests that miRNAs in the cancer cells are more susceptible to degradation, due to its interaction with a non-coding 3′UTR. This non-coding component of mRNA may be used retrospectively to modulate miRNA activities.

## Introduction

Mature miRNAs are single-stranded RNAs of approximately 21 nucleotides in length. In the cytoplasm, mature miRNA and Argonaute proteins make up the RNA-Induced Silencing Complex (RISC) and function by complementary base-pairing with the 3′-untranslated regions (3′UTR) of target mRNAs [1], [2]. As a result, mRNA translation is repressed, and mRNA stability is also endangered [3]. Using computational algorithms it was predicted that miRNAs regulate about 30% of human genes [4], but a recent inspection of human 3′UTR has shown that more than 60% of protein-coding genes maintain conserved target sites for miRNA recognition [5]. The regulatory role of miRNA has been extensively studied in various fundamental processes such as development [6], [7], differentiation [8]–[10], cell proliferation [11], [12], apoptosis [13], [14], cell cycle [15], [16], and immune responses [17], [18]. Timing of gene regulation is important in these processes, and the 3′UTR of mRNAs have been found to contain more than one target site recognized by the same miRNA [19], [20]. Proteomics studies have shown that a single miRNA impacts translation of hundreds of mRNAs [21], [22]. In these studies, most 3′UTRs of these mRNAs harbor target sites that match the seed region of the miRNA, suggesting that miRNAs with similar seed regions may have overlapping functions. In addition, there is evidence demonstrating that one miRNA can regulate expression of multiple genes of related function in order to fine tune cell activities [23]. Thus, miRNAs that target the 3′UTR of a particular mRNA may also target a set of mRNAs with similar function. Although some studies and models have suggested simple regulation of genes by a miRNA, there is accumulating evidence that multiple miRNA molecules may regulate a particular gene. Along with this hypothesis, we have previously developed a PCR method to screen miRNAs that potentially bind to a specific 3′UTR [24]. In this study, we investigated different miRNAs that regulate the 3′UTR of a gene. A fragment of versican 3′UTR was expressed in an *in vitro* cell model, and its effect on miRNAs levels and cell activities were examined. The role of the 3′UTR other than being a cis-element of the mRNA was thus unveiled.

## Results and Discussion

### Expression of versican 3′UTR reduces cell proliferation and tumor growth

**Figure 1:**
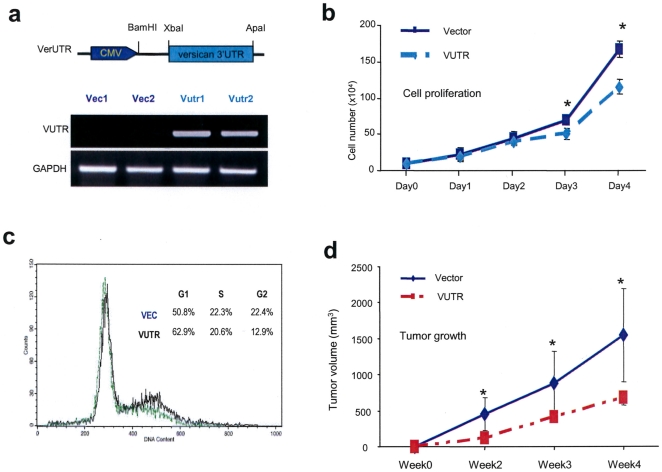
Expression of versican 3′UTR reduces cell proliferation and tumor growth. (a) Mouse breast carcinoma cells 4T1 was transfected with a fragment of versican 3′UTR (700 bp) or a control vector. Pooled cells were obtained. RNA was isolated from the pooled cells and the expression of 3′UTR was confirmed by RT-PCR. (b) Cell proliferation assays were performed in cells transfected with the control vector or the 3′UTR in low serum conditions (1.5% FBS) by cell counting after trypan blue staining. *, P<0.05. Error bars indicate SD (n = 5). (c) Cell cycle analysis by FACS confirmed that the vector-transfected cells had greater population of G2/M cells than the VerUTR-transfected cells. Representative data is shown. (n = 3). (d) The cells were injected subcutaneously into BALB/c mice. Tumor size was recorded weekly and tumor growth curve was obtained for a period of four weeks. Asterisks indicate significance. *, P<0.05. Error bars indicate SEM (n = 3).

An expression construct was generated to study the function of 3′UTR. The conserved region of versican 3′UTR (2285–3000 bp, Genebank access number, NM_001126336.1) was cloned and inserted in front of a CMV promoter producing the construct VerUTR (Figure 1a). The construct was stably expressed in a mouse breast carcinoma cell line, 4T1, and its expression was confirmed by RT-PCR. This cell line was chosen because of its compatibility with BALB/c mice without rejection of transplanted cells by the host′s immune system. Injecting these cells into the mice represents an isogenic relationship between the host and the tumor cells, and allows the studies of molecularly modified tumor cells. Tumor growth and metastatic invasion induced by the 4T1 cells closely mimic human breast cancer progression, and is an established animal model for stage IV human breast carcinoma.

To examine the effect of the expression construct VerUTR on 4T1 cells, we analyzed proliferation rates of the cells. Cells transfected with VerUTR and cells transfected with a control vector were cultured in low serum medium. We observed reduced proliferation in the VerUTR cells as compared with cells transfected with an empty vector (Figure 1b). Cell cycle was analyzed by staining cells with propidium iodide. FACS analysis demonstrated that there were approximately twice as many ‘control’ cells in the G2/Mitosis phase than among the VerUTR population (Figure 1c). On the other hand, more VerUTR cells than control cells were stalled in the G1 phase where cells were not committed to DNA synthesis.

In tumor formation experiments, VerUTR- and vector-transfected cells were subcutaneously injected into BALB/c mice. Two weeks following the injection, there was a noticeable difference in tumor size (Figure 1d). Tumors generated by cells transfected with VerUTR were consistently smaller than tumors generated in the control group. Statistical analysis showed that the difference in tumorigenesis was significant two weeks post-injection. Colony formation assays also revealed similar results (Supplementary Information, Figure S1). We reasoned that exogenous expression of the versican 3′UTR interfered with and arrested miRNA functioning, which in turn could relieve potential targets of these miRNAs. The results also suggest that these miRNAs are important in supporting cell proliferation and tumor growth. Thus, these miRNAs may function together during oncogenesis.

**Figure 2:**
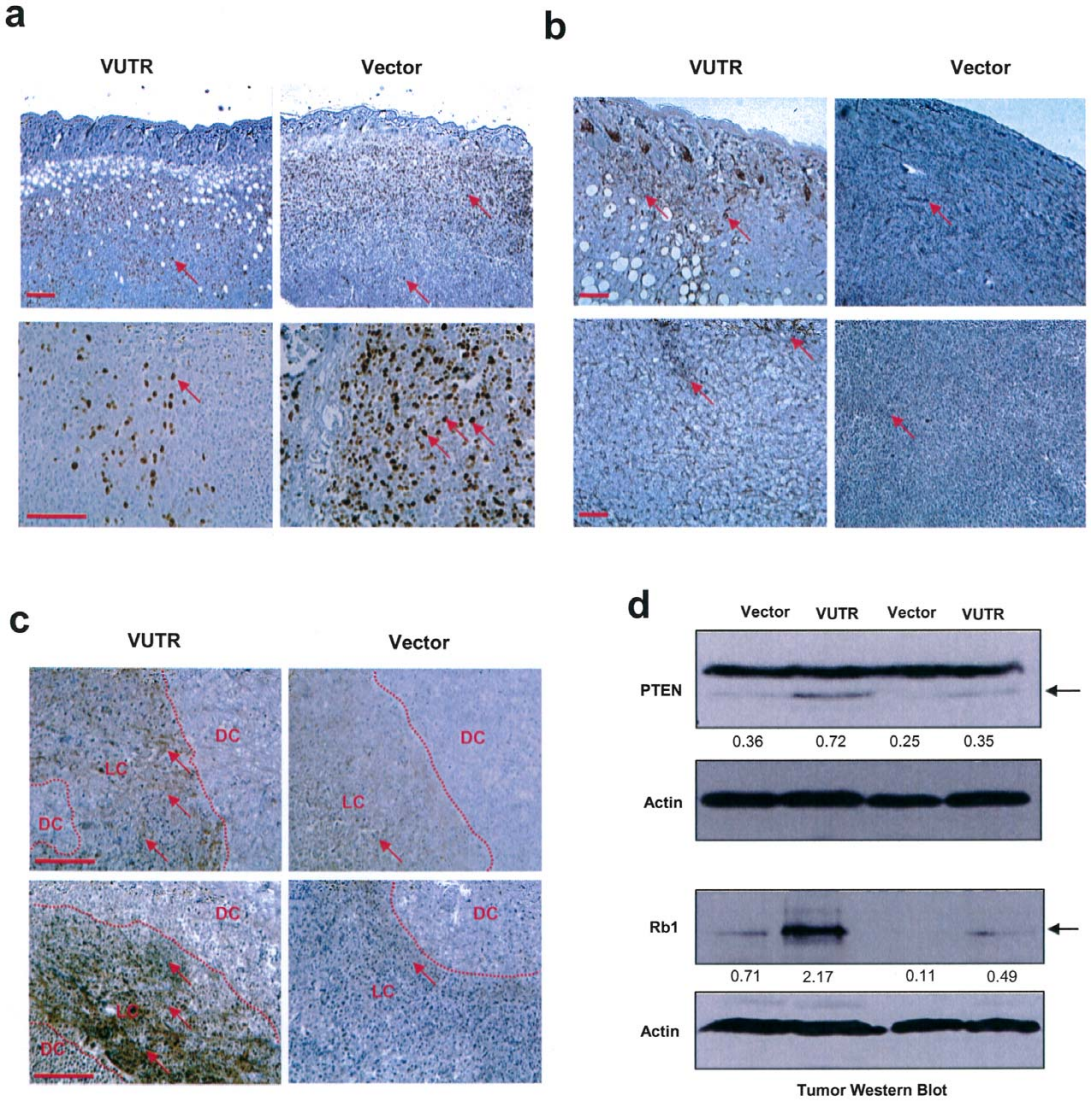
Expression of versican 3′UTR reduces Ki67 but enhances PTEN expression. (a) The tumors were sectioned and stained for Ki67 expression. The vector tumors showed higher levels of Ki67 staining (arrows). scale bars, 100 µm. (b) The tumor sections were stained for PTEN expression. Increased PTEN expression was detected in the 3′UTR tumors. scale bars, 100 µm. (c) PTEN expression was higher in the areas of live cells (LC) adjacent to the areas of dead cells (DC), which was more evident in the versican 3′UTR tumors than in the vector tumors. scale bars, 100 µm. (d) The tumor lysates were subject to western blot analysis, probed with anti-PTEN, anti-Rb1, and anti-actin antibodies. There was significant elevation of Rb1 and PTEN levels in the 3′UTR tumors compared with the vector control.

The tumors were dissected, fixed and then sectioned for immunohistochemistry. Staining with Ki67, a cell proliferation marker, showed many Ki67-positive cells in tumors formed by vector-transfected cells, especially at the peripheral edges of tumors where cells proliferate (Figure 2a). Nevertheless, there was detectable staining of Ki67 in the tumors formed by the VerUTR-transfected cells. The staining appeared to be in small patches throughout the tumor section, and it is possible that the cells may be expressing low levels of VerUTR in the pooled cell population. High magnification photos displayed a greater number of Ki67-positive cells in the control vector tumors than in the VerUTR tumors. This indicates that the cells transfected with control vector were proliferating at a higher rate, which resulted in faster tumor growth.

Tumor sections were examined for expression of two negative cell cycle regulators, PTEN and Rb1, which could affect the proliferation of the VerUTR-transfected cells. Staining with anti-PTEN antibody showed a strong elevation of PTEN expression ubiquitously throughout the tumor sections formed by the VerUTR-transfected cells (Figure 2b). Tumors formed by control cells displayed little expression of PTEN in patchy regions of the tumors. Interestingly, there were tumor cells expressing PTEN in the core of the VerUTR tumors, an area usually packed with cell debris and surviving cells. It is possible that impaired miRNA function reduced cell proliferation but enhanced cell survival. In addition, PTEN levels were higher in live cells adjacent to dead cell areas, and this was more evident in VerUTR tumors than in the vector tumors (Figure 2c). However, the differences in tumor sizes could not be ruled out as the cause of this observation. Smaller VerUTR tumors may be supplied with comparatively more adequate and sufficient small blood vessels. Tumor sections were also stained with Masson's trichrome stain for collagen visualization (SI Figure S2). The smaller VerUTR tumors were displayed less collagen staining when compared to ‘control’ tumors, which were of significantly larger size. The difference in abundance due to tumor size did not change the collagen distribution.

Tumor tissues were grinded and lysed, and the expression of PTEN and Rb1 were further examined by western blot analysis (Figure 2d). Compared with tumors formed by the control cells, tumors formed by the VerUTR-transfected cells exhibited increased expression of PTEN and Rb1. The elevated expression of these two negative cell cycle regulators could have synergistically reduced cancer cell proliferation and thus tumor growth.

Since miR-199a-3p has been shown to target versican and fibronectin [24], tumor sections were stained with anti-versican and anti-fibronectin antibodies. The staining showed that there was indeed an elevation of versican and fibronectin expression in the tumors formed by VerUTR-transfected cells. These results suggest that the function of miR-199a-3p was likely impaired (SI Figure S3). Transfection of VerUTR into the cells facilitated increased translation of versican and fibronectin. It is already known that versican isoforms V0 and V1 are expressed in the early stages of tissue development, while the isoform V2 is expressed in mature tissues [25], [26]. We have previously demonstrated that the V1 isoform can enhance cell proliferation, induce cell transformation, and tumor formation, while the V2 isoform can inhibit cell proliferation and induce apoptosis [27], [28]. Although it is not known which isoform is expressed in breast cancer, our results suggest that expression of V2 isoform may be up-regulated, resulting in decreased cell proliferation and tumor growth.

### Rb1 is targeted by miR199a-3p and miR-144

Currently, miR-199a-3p is the only miRNA that has been shown to regulate versican expression by binding to its 3′UTR. However, versican 3′UTR has been shown to interact with a number of other miRNAs [24]. By computational algorithms, a number of genes that are both potential targets of these miRNAs and are negative regulators of cell cycle were obtained. A few of these genes are of particular interest because of their targeting by multiple versican-bound miRNAs, including Retinoblastoma1. Rb1 is one of the well-known tumor suppressors studied and primary cancer occurs when Rb1 protein is lost or dysfunctional [29]. Rb1 plays the role as a negative cell cycle regulator by postponing cells from passing G1 inter-phase [30]. Expression of Rb1 results in reduced cell proliferation and tumor growth.

**Figure 3:**
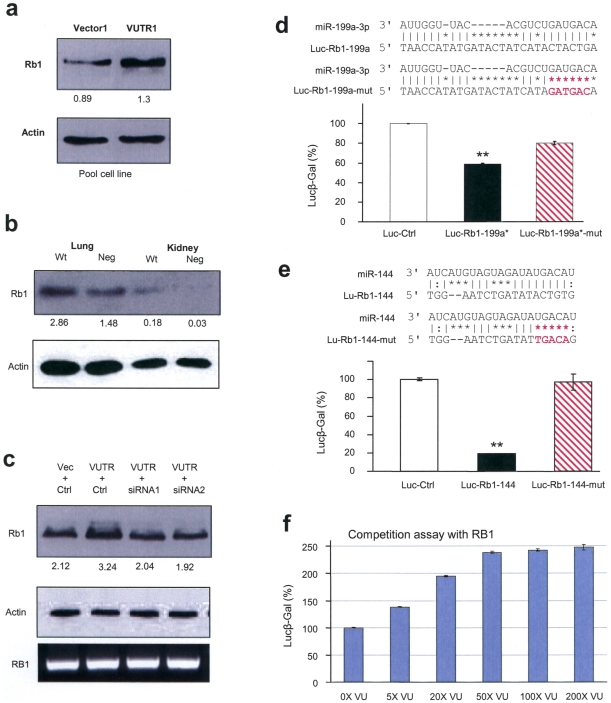
Rb1 expression is regulated by miR-199a-3p and miR-144. (a) Cell lysate from the 3′UTR- or vector-transfected cells was analyzed on western blot for Rb1 expression. Rb1 levels were elevated in the VerUTR cells. (b) Lysates prepared from lung and kidney of VerUTR transgenic and wildtype mice were analyzed by western blot probed with anti-Rb1 antibodies. Increased expression of Rb1 was detected in the organs from the VerUTR transgenic mice. (c) 4T1 cells were co-transfected with VerUTR and siRNAs against 3′UTR or a control sequence. The negative control was performed by co-transfecting the cells with the vector plasmid and the control sequence. Cell lysates were prepared from transfected cells and subject to western blot analysis probed with anti-Rb1 and anti-actin antibodies. Decreased expression was observed in the cells co-transfected with VerUTR and siRNAs as compared with that co-transfected with VerUTR and the control sequence. (d) The Rb1 3′UTR containing the potential target site of miR-199a-3p was cloned into a luciferase vector generating a construct luc-Rb1-199a. The binding site was mutated (in red) generating luc-Rb1-199a-mut. U343 cells were co-transfected with miR-199a-3p and the luciferase constructs. Luciferase activity assays showed that miR-199a-3p repressed luciferase activities luc-Rb1-199a. The repression was abolished when the mi-199a-3p binding site was mutated. **, p<0.0001. Error bars, SD (n = 3). (e) A fragment of Rb1 3′UTR containing the binding site for miR-144 was cloned and mutated (in red), generating luc-Rb1-144 and luc-Rb1-144-mut, respectively. U343 cells were co-transfected with miR-144 and the luciferase constructs. Luciferase activity assays showed that mutation of miR-144 binding site removed the translational inhibition exerted by miR-144. **, p<0.0001. Error bars, SD (n = 3). (f) Luciferase reporter vector harboring the Rb1 3′UTR was co-transfected with VerUTR construct in increasing amounts of plasmid DNA complemented with the control vector in U343 cells. Increased abundance of VerUTR allowed translation of luciferase construct, resulting in elevation of luciferase activities.

Cell lysates prepared from cells transfected with VerUTR or the control vector were analyzed for Rb1 expression by western blotting. Probing with anti-Rb1 antibody showed that there was an elevated expression of Rb1 in the VerUTR cells (Figure 3a). Primary lung and kidney tissues were collected from the VerUTR transgenic and wildtype mice, and similar results were observed (Figure 3b). It appears that presence of the 3′UTR promoted Rb1 expression. To confirm this result, a rescue experiment was performed by employing siRNA against the 3′UTR. 4T1 cells were transfected with the 3′UTR together with siRNAs against the 3′UTR or with a control sequence. As a negative control, another set of 4T1 cells were transfected with the vector and the control sequence. Transfected cells were then analyzed for Rb1 expression. Increased Rb1 levels were observed in the 3′UTR-transfected cells, reinforcing previous findings (Figure 3c). Transfection with the addition of siRNAs successfully knocked down the expression of Rb1 to the expression level comparable to that of the negative control. This suggests that removal of 3′UTR can readily reduce the expression of Rb1. RNA of a duplicated set of transfected cells was isolated and subjected to RT-PCR analysis. No significant differences in Rb1 mRNA levels were detected. Results of these experiments strongly related the translational efficiency of Rb1 mRNA with the presence of 3′UTR.

Two miRNAs (miR-199a-3p and miR-144) that have been shown to interact with versican 3′UTR [24] were hypothesized to inhibit Rb1 translation (SI Figure S4a). To validate this potential regulation, luciferase experiments were performed. The potential binding sites of miR-199a-3p were found on Rb1 3′UTR (nucleotide 654-675, GeneBank Accession No. NM_000321.2). The miR-199a-3p target site was highly conserved in human and mouse genome, suggesting its importance in regulating Rb1 translation. Therefore, a region of Rb1 3′UTR containing this binding site was cloned into a luciferase reporter vector. Another luciferase construct with mutated nucleotides located in the miRNA-binding site was generated (Figure 3d). The presence of a mutated binding site alleviated luciferase activity from translational inhibition by miR-199a-3p and restored luciferase activity by 20%. Rb1 was a potential target of miR-144, another miRNA that binds to versican 3′UTR [24]. The potential binding site of miR-144 on Rb1 3′UTR (nucleotide 674-695) was found to be close to the miR-199a-3p target site. Moreover, this miR-144 site is highly conserved in the human and mouse genome (SI Figure S4). Strong conservation of this region in the Rb1 3′UTR implied its importance in miRNA interaction. This region of Rb1 3′UTR including the potential miR-144 binding site or its mutated version were cloned to generate luciferase constructs. The construct harboring the miR-144 target sequence significantly repressed luciferase activity compared with the control vector harboring a nonrelated fragment (Luc-Ctrl) or the mutated construct (Figure 3e). This suggests direct targeting of Rb1 by miR-144.

Instead of performing luciferase assays by transfecting miRNA mimics, cells were co-transfected with a luciferase construct harboring Rb1 3′UTR and increasing concentrations of versican 3′UTR (Figure 3f). As increasing quantities of 3′UTR was co-transfected with luciferase constructs, more miRNAs bound to the 3′UTR and thus luciferase showed increased activity. However, luciferase activities tended to reach plateau when 50-folds of the 3′UTR were co-transfected. This suggests that translation of endogenous Rb1 can potentially be up-regulated by 2.5-fold without changing the transcription of the gene. Based on the results of Rb1 targeted by miR-144 and miR-199a-3p, it is anticipated that other miRNAs targeting Rb1 could function in the similar fashion. In mice, miR-199a-3p has another target site located nearby the first confirmed target site (SI Figure S5a). A known tumor suppressor-like miRNA, miR-16, binds to versican 3′UTR and has a hypothetical target site in Rb1 mRNA. However, it does not seem to be the determining factor in this breast cancer model.

### miR-144 and miR-136 repress PTEN translation

**Figure 4:**
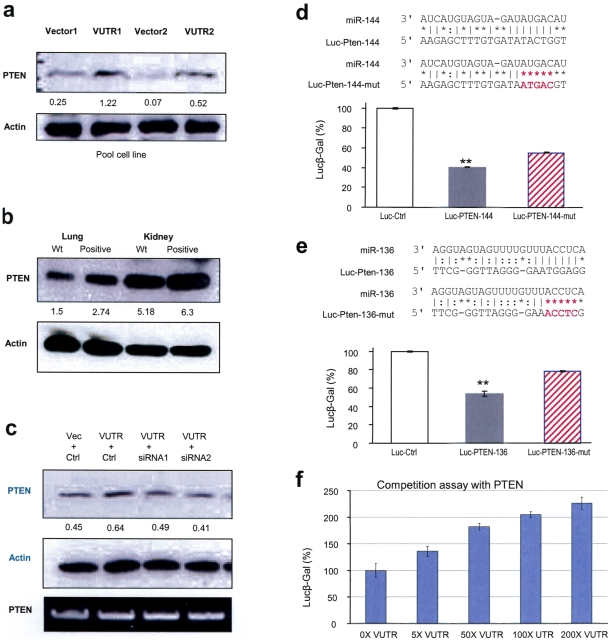
Targeting of PTEN by miR-144 and miR-136. (a) Cell lysates prepared from the VerUTR and control cells were analyzed on western blot probed with anti-PTEN antibody. Increased PTEN expression was detected in the VerUTR cells, while protein loading is equal as exhibited by actin staining. (b) Lysates prepared from lung and kidney of VerUTR transgenic and wildtype mice were probed with anti-PTEN antibody by western blot analysis. Increased PTEN expression was observed in the organs of the VerUTR transgenic mice. (c) 4T1 cells were transiently transfected with VerUTR and siRNAs against 3′UTR or control sequence. As a negative control, 4T1 cells were also transfected with control vector and control sequence. Lysates were prepared from transfected cells and analyzed by western blot probed with anti-PTEN and anti-actin antibodies. Decreased PTEN expression was observed when the VerUTR was co-transfected with siRNAs against VerUTR. (d) Hypothetical targeting site by miR-144 in the 3′UTR of PTEN was cloned and mutated (in red), generating two constructs luc-PTEN-144 and luc-PTEN-144-mut. In luciferase activity assays, U343 cells were co-transfected with luc-PTEN-144 or luc-PTEN-144-mut with miR-144. Luciferase activity of mutated construct was higher than that of the construct expressing original 3′UTR. **, p<0.001. Error bars, SD (n = 3). (e) miR-136's potential targeting sequence in the 3′UTR of PTEN was cloned and mutated (in red), generating two constructs luc-PTEN-136 and luc-PTEN-136-mut. In luciferase activity assays, U343 cells were co-transfected with luc-PTEN-136 or luc-PTEN-136-mut with miR-136. Repression on luciferase activity was removed when miR-136 targeting site was mutated. **, p<0.001. Error bars, SD (n = 3). (f) Luciferase reporter vector harboring the PTEN 3′UTR was co-transfected with VerUTR construct in increasing amounts of plasmid DNA complemented with the control vector in U343 cells. Increased rations of VerUTR bound more endogenous miRNAs and thus freeing the translation of luciferase protein, resulting in higher levels of luciferase activities.

Another gene of interest was Phosphotase and Tensin Homolog, or PTEN. PTEN has been shown to function as a tumor suppressor, where loss or mutation of this gene leads to cancer predisposition [31]. Similar to Rb1, PTEN is also known as a negative cell cycle regulator by arresting cells in G1 phase [32]. Increased expression of PTEN resulted in decreased cell proliferation. To confirm these results, pooled cells transfected with VerUTR or the control vector were lysed and probed with anti-PTEN antibody for western blot analysis. We observed an elevation of PTEN expression in the VerUTR-transfected cells (Figure 4a). When primary tissues were examined for PTEN expression, similar results were obtained but were less significant in kidney tissues (Figure 4b). Staining of PTEN in the primary lung tissues also yield a similar result, while expression of other proteins were not influenced (not shown). To confirm that VerUTR could antagonize PTEN for miRNA binding, we performed knockdown experiments using siRNA against versican 3′UTR and detected an elevation of PTEN expression in the VerUTR-transfected cells (Figure 4c). Knockdown of VerUTR by siRNA abolished the increased PTEN expression to the same level seen in cells transfected with the control vector.

Luciferase experiments were performed to validate the targeting of PTEN by proposed miRNA candidates (SI Figure S4b). miR-144, which potentially targets Rb1 as indicated above, was also predicted to target PTEN. The miR-144 binding site in the 3′UTR of PTEN (nucleotides 2906–2925 bp, GeneBank Accession No. NM_000314.4) was highly conserved between human and mouse genomes and was cloned into a luciferase vector, producing Luc-Pten-144 (SI Figure S4b). The seed sequence matching site on the PTEN 3′UTR was mutated, producing Luc-Pten-144-mut (Figure 4d). Luciferase activity of Luc-Pten-144 was repressed by miR-144 but could be restored when the target sequence was mutated (Figure 4d).

By computational predictions, another versican-bound miRNA, miR-136, could potentially target PTEN through three binding sites on the PTEN 3′UTR (nucleotides 289–311; 490–511; and 2759–2781). Two of these three sites are highly conserved in the human and mouse genomes (SI. Figure S4b). It is possible that a 3′UTR could harbor multiple sites targeted by the same miRNA, and this has been shown by us in the study demonstrating that CD44 3′UTR is regulated by miR-328 through multiple target sites [20]. A segment of PTEN 3′UTR harboring one of the binding sites (nucleotides 2759–2781) and its mutated version were cloned into a luciferase vector, producing Luc-Pten-136 and Luc-Pten-136-mut, respectively (Figure 4e). miR-136 significantly repressed luciferase activity of Luc-Pten-136, and the repression was almost abolished when the target sequence was mutated.

In luciferase competition assays, increasing amounts of VerUTR were co-transfected with a luciferase construct containing PTEN 3′UTR (Figure 4f). As increasing amounts of VerUTR were transfected to antagonize endogenous miRNAs, luciferase activity elevated respectively, signifying increasing translation of luciferase. Although 200-folds of VerUTR were used in the transfection, luciferase activity was 2.3-fold greater than its starting level of activity but its maximum level was not reached. It is possible that many miRNAs regulate PTEN or that the presence of miRNAs that target PTEN was very abundant in the cells. As a consequence, VerUTR could only partially regulate PTEN expression. Besides miR-144 and miR-136, two other miRNAs, miR-16 and let-7 miRNA, could potentially interact with versican 3′UTR and have hypothetical target site on the PTEN mRNA (SI Figure S5b). The absence of these target sites from the human genome suggests that it is unlikely that they regulate PTEN expression.

### miRNA level is affected in the presence of 3′UTR

In this study, we expected to interfere with miRNA targeting by expressing 3′UTR as a decoy. We next examined how 3′UTR affected miRNA expression *in vitro* and *in vivo*. We have chosen to focus on the expression of miR-199a-3p and miR-136 because miR-144 is an erythroid lineage-specific miRNA [33]. The expression of miR-199a-3p and miR-136 were detected in both cells and several primary tissues but miR-144 was not found using RealTime-qPCR (results not shown).

**Figure 5:**
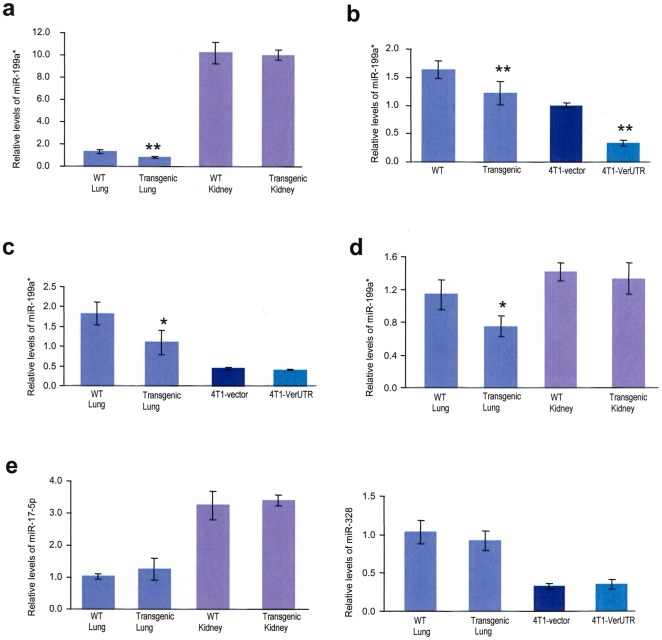
Expression of VerUTR affects miRNA levels. RNAs from pooled cell lines and primary tissues of the VerUTR transgenic and wild type mice were isolated. By Real-time PCR, relative quantities of miRNAs were determined and compared with the use of miRNA-specific primers. Primary organs from twelve mice at the age between four to six months were analyzed in these experiments. Quantitative values of miRNA levels were normalized with U6 RNA levels for comparison. Representative data is shown. (a) In the lung tissues, the levels of miR-199a-3p were about 39% lower in the VerUTR transgenic mice compared with that in the wildtype. In contrast, there was no significant difference between transgenic and wildtype kidney tissues. **, p<0.01. Error bars, SD (n = 3). (b) The levels of miR-199a-3p were compared between primary tissues and cancer cell lines. There is a 29% reduction in miR-199a-3p levels in the liver tissues of transgenic mice compared with that of wildtype. In the 4T1 cell line, cells transfected with VerUTR showed 73% reduction of miR-199a-3p compared with cells transfected with the control vector. **, p<0.01. Error bars, SD (n = 4). (c) Levels of miR-136 were significantly lowered in the lung tissues of transgenic mice compared with that of wildtype by 40%. There was no significant difference in miR-136 levels between the cells transfected with either VerUTR or the control vector. *, p<0.05. Error bars, SD (n = 3). (d) Expression of miR-136 in lung and kidney tissues of the same mice was inspected. *, p<0.05. Error bars, SD (n = 3). (e) Expression of unrelated microRNAs miR-17-5p and miR-328 were analyzed to ensure that the subjects are comparable and the levels of other miRNAs were not affected.

In our previous studies, miR-199a-3p was found to regulate two extracellular matrix proteins, versican and fibronectin [24], and it has also been shown to be important during development [34]. Expression of miR-199a-3p was analyzed in the primary lung and kidney of VerUTR transgenic and wildtype mice. Reduction in miRNA expression was observed in these primary tissues. There was a 39% reduction in miR-199a-3p expression in lung tissues but no significant changes were found between the transgenic and wildtype kidney tissues (Figure 5a). We reasoned that different tissues might exhibit different levels of miR-199a-3p expression, but more importantly, different tissues might respond differently to the presence of 3′UTR. Using primary liver tissues as another example, we detected a 29% reduction in miR-199a-3p expression in the presence of 3′UTR (Figure 5b). In addition, cells transfected with 3′UTR lost 79% of its miR-199a-3p compared with the control cells.

Expression of miR-136 was also inspected in the primary tissues and cells expressing VerUTR. In the primary lung tissues, there was a 40% significant reduction in miR-136 expression, while a significant difference between breast cancer cells transfected with VerUTR and the control vector was not detected (Figure 5c). Similarly, there was little difference in miR-136 levels in the kidney of VerUTR transgenic and wildtype mice (Figure 5d). Expression of two unrelated miRNAs, miR-17-5p and miR-328, were examined using the same samples. No significant difference was observed (Figure 5e).

Notably, the levels of miR-199a-3p and miR-136 were consistently reduced in lung tissues but not kidney tissues. The abundance of miRNA was not likely the reason for this because miR-136 levels were similar in both lung and kidney (Figure 5d). Clearly, other factors beyond expression levels, such as cell types, are involved in 3′UTR-mediated miRNA instability in these tissues. A previous study suggested that imperfect miRNA targeting is a better decoy than perfect targeting [35]. Naturally evolved 3′UTR contains imperfect binding sites for miRNAs, and thus was expected to be able to antagonize miRNAs efficiently. In addition, we found that antagonizing miRNA with non-coding 3′UTR sequences led to miRNA instability. Human Argonaute 2 (Ago2) is part of RISC, the miRNA functional unit, and has been shown to mediate endonucleolytic cleavage of mRNA when there is perfect complementarity [36]. This could be a good model to study mechanisms involved in miRNA instability. Our results showed a general loss of miRNA abundance by 30% depending on the types of tissues, while other miRNAs were not affected because their targets were translated at the same efficiency (Figure 5e). Although we only examined the abundance of two miRNAs, other miRNAs that bind to 3′UTR are likely to be affected as well. Transient or stable transfection of 3′UTR into breast cancer cells both reduced the cellular levels of miR-199a-3p, suggesting that the degradation process is immediate and can be prolonged.

We also observed that cancer cells tended to lose miRNA by nearly two-fold more than primary tissues examined. It is well accepted that genomic instability is common during cancer progression. Due to genomic alterations, miRNA expression is dysregulated [37], and non-coding transcripts are generated during this process. Binding of miRNAs to these transcripts may have also contributed to the global reduction of miRNA levels and functions. Currently, the mechanisms of miRNA degradation are not as well understood as its biogenesis. An exoribonuclease found in *Arabidopsis* named Small RNA Degrading enzyme 1, or SDN1, is one of the few enzymes reported to degrade miRNA [38]. Further studies are needed to investigate the underlying mechanisms of miRNA degradation.

Different approaches to regulate miRNA activity have been tested, including decoys [39], sponges [40], locked nucleic acid (LNA) [41], and antagomir [42]. Most of them are chemically modified antisense oligonucleotides and hence usually lead to miRNA antagonism but not miRNA degradation. Since they are meant to antagonize specific miRNAs, they are often designed with perfect seed region match and multiple repeats. Therefore, they are very effective against a specific miRNA or a miRNA family such as let-7. However, there is evidence that multiple miRNAs can target a gene, and antagonizing one miRNA may not relieve enough translational repression exerted by other miRNAs. miRNA activity relies on a threshold level of expression [43], and certain miRNAs may not be expressed abundantly in certain cell types. In order to be able to efficiently control the translational efficiency of a gene, knowledge of the detailed mechanisms of miRNA regulation is required. Studies on 3′UTR allow us to experimentally validate hypothetical miRNAs that are based on its seed region match with the conserved target sites. Assays for validation include PCR for identification of 3′UTR binding miRNAs, real-time PCR for detection of miRNA instability, and luciferase reporter assays for confirmation. Study of the 3′UTR/miRNA interactions will help in identification of the best potential miRNA candidates, whereas combining multiple miRNAs will likely result in more robust biological effects.

With the discovery of miRNAs, various cis-elements that enable miRNA-mediated translational control are being discovered in 3′UTRs. Its secondary structure formed by regions flanking target sites determines miRNA accessibility [44]. A uridine-rich region in the 3′UTR can be bound by Dead End 1 (Dnd1), which prohibits miRNA from associating with their target site [45]. The AU-rich element in the 3′UTR can potentially lead to translation activation by miRNAs [46]. Our study suggests that while 3′UTR enables translational regulation by miRNA, it also regulates miRNA activity by binding to miRNAs. This means that transcription of a gene will relieve translational suppression on its own mRNA and others that are regulated by the same miRNA. Because miR-199a-3p regulates translation of both versican and fibronectin, transcription of versican will share the load of suppression and allow increased translation of both versican and fibronectin [24]. Therefore, results from this study suggest that transcription of a gene may affect translation of another gene through miRNA interactions. In terms of biological function, this study shows that feed-forward and feedback mechanism are both possible by versican transcription. Versican V1 and V2 isoforms are known to exhibit opposite functions on cell proliferation [27]. When anti-proliferative versican V2 is transcribed, synergistic action of versican V2, Rb1, and PTEN may work strongly against proliferation. On the other hand, when proliferation-favored versican V1 is transcribed, increased translation of Rb1 and PTEN, if both mRNAs are present at high levels, will work against the proliferative property of V1 but may keep its function in cell adhesion. This mechanism can be particularly important for cell survival where nutrients are scarce, as other miRNAs, e.g. miR-143 [47], can interact with the 3′UTR of both isoforms.

**Figure 6:**
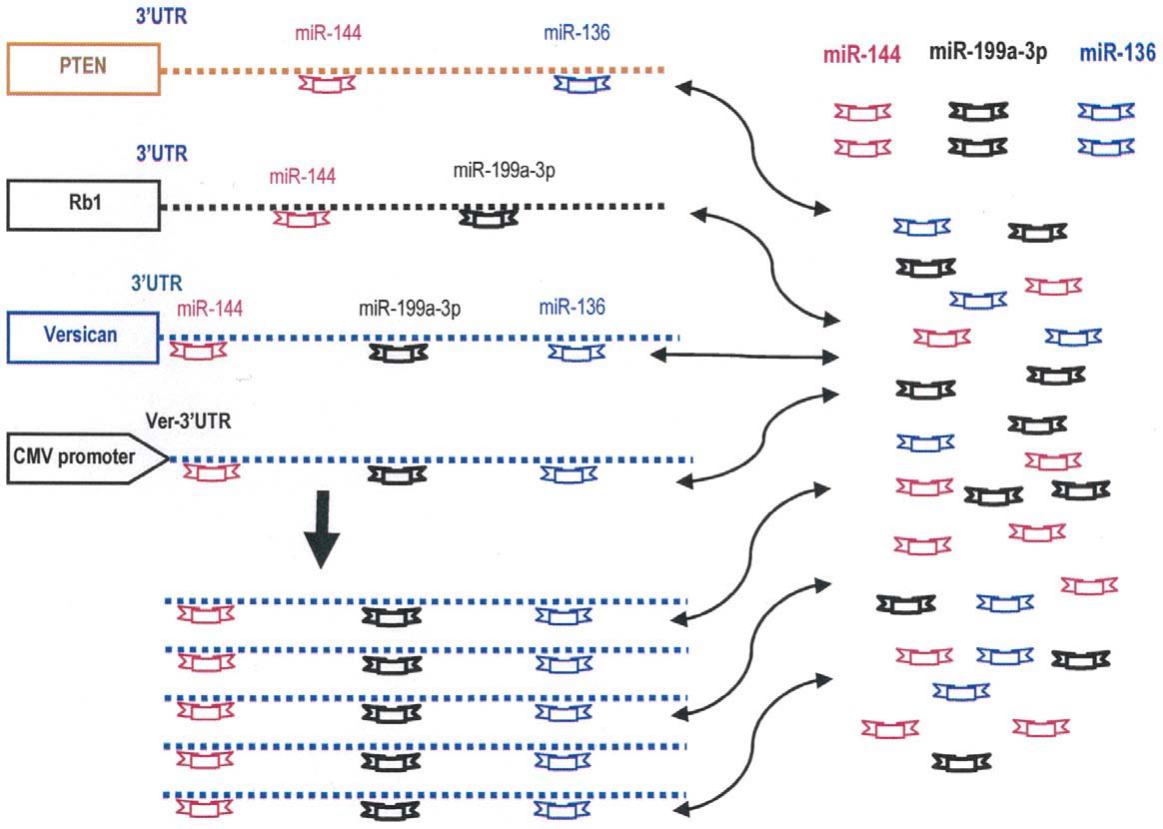
Relationships of miRNA interaction with 3′UTR. Computational analysis showed that miR-199a-3p, miR-144, and miR-136 potentially targeted versican 3′UTR. Overexpression of versican 3′UTR would attract endogenous miR-199a-3p, miR-144, and miR-136. As a consequence, the mRNAs of versican, Rb1, and PTEN are relieved to proceed with translation.

The 3′UTR seems to be a mediator of miRNA function, but it also acts as a regulator in modulating miRNA function. We found that versican 3′UTR is targeted by multiple miRNAs, which have common targets such as Rb1 and PTEN. Expression of non-coding 3′UTR results in up-regulation of Rb1 and PTEN, which in turn reduces cell proliferation and tumor growth. These results are summarized in Figure 6, and depict schematically that overexpression of versican 3′UTR driven by a CMV promoter could result in binding of miR-144, miR-199a-3p, and miR-136. As a consequence, diminished interactions of these miRNAs with the mRNAs of vesican, Rb1, and PTEN may occur, which in turn may relieve these mRNAs for translation. Our study of 3′UTR reveals the existence of a RNA signaling network that has previously been unknown. We anticipate that studies of 3′UTR will aid in identifying targets of miRNA and its application in amplifying the effect on coding transcripts by regulating miRNA activities.

## Materials and Methods

### Generation of 3′UTR constructs and transgenic mice

The versican 3′UTR expressing construct, VerUTR, that transcribes conserved regions of versican 3′UTR, was previously described [24]. A luciferase reporter vector (pMir-Report; Ambion) was used to generate the luciferase constructs. The 3′UTR of Rb1 was cloned using forward primer, huRB1-SacI, and reverse primers, huRB1-miR144MulI and huRB1-miR199a*MulI, by PCR. The PCR products were digested with SacI and MluI and then inserted into a SacI and MluI-digested pMir-Report Luciferase plasmid, to obtain luciferase constructs, Luc-RB1-144 and Luc-RB1-199a. Primers used in this study are listed in the SI Figure S6. Mutant constructs were generated with PCR by the same forward primer but different reverse primers, huRB1-miR144MulI-mut and huRB1-miR199a*MulI-mut. After double restriction enzyme digestion by SacI and MluI, both fragments were ligated with pMir-Report vector opened with SacI and MluI.

PTEN luciferase constructs were generated similarly. The 3′UTR of PTEN was cloned by PCR using forward primer, huPTEN-SacI, and two reverse primers, huPTEN-miR144MulI and huRB1-miR136MulI. The PCR products were double digested with SacI and MluI and ligated with SacI and MluI-opened pMir-Report Luciferase plasmid, to obtain luciferase constructs, Luc-PTEN-144 and Luc-PTEN-136. Two mutant constructs were generated with PCR by reverse primers, huPTEN-miR144MulI-mut and huRB1-miR36MulI-mut. After restriction enzyme digestion by SacI and MluI, both fragments were ligated to pMir-Report vector opened with SacI and MluI.

To serve as a negative control, a non-related sequence was amplified from the coding sequence of the chicken versican G3 domain as previously described [23]. No miRNAs are expected to bind to this fragment as it is in the coding region.

The VerUTR transgenic mice were generated by microinjecting a DNA fragment excised from versican 3′UTR expressing construct VerUTR into male pronuclei of C57BL/66CBA F2 mouse zygotes. Details of generation and genotyping methods were previously described [24]. Tissue harvest and analysis have been approved by the Animal Care Committee of Sunnybrook Research Institute (Animal Use Protocol: 09-076), Ontario, Canada.

### Tumor Formation Assay and Immunohistochemistry

Six-week-old BALB/c mice received subcutaneous injections of VerUTR- and vector-transfected 4T1 cells (5×10^5^ cells). Tumor growth was monitored weekly and the sizes were recorded using a caliper by determining the length (L) and width (W), where V =  (L×W^2^)/2. Tumors were retrieved by the end of fourth week, fixed in formalin, and sectioned. After antigen retrieval, sections were blocked with 10% goat serum and incubated with primary antibody against PTEN (clone 6H2.1, Millipore), or Rb1 (ab32199, ABCAM) in TBS containing 1% bovine serum albumin (BSA) overnight. The sections were washed and labeled with biotinylated secondary antibody, followed by avidin conjugated horse-radish peroxidase provided by the Vectastain ABC kit (Vector, PK-4000). DAB chromogenic reaction was performed according to manufacturer's protocols. The slides were subsequently counter-stained with Mayer's Hematoxylin and mounted.

### Cell Proliferation Assay and Cell Cycle Analysis

Cells were seeded onto 6-well plates at a density of 5×10^4^ cells/well in 1.5% FBS-containing medium. Cell number was counted by trypan blue staining daily for a period of four days. Cells were then centrifuged at 1200 rpm for 3 minutes after initial fixation with 70% cold ethanol and following washes by PBS. In the final step, 1×10^6^ cells were resuspended in 1 ml PBS with the addition of 100 µl ribonuclease (100 µg/ml, Sigma) and 400 µl propidium iodide (50 µg/ml, Sigma). After leaving the samples at room temperature for five minutes, they were acquired by a FACScan flow cytometer (BD Biosciences), and data was analyzed using CellQuest software.

### Luciferase Activity Assay

Luciferase activity assay was performed using a dual-luciferase reporter system developed by Promega (E1960) using the methods described by us [48]. In short, U343 cells were seeded onto 24-well tissue culture plates at a density of 3×10^4^ cells/well in 10% FBS containing medium for 24 h. Cells were co-transfected with the luciferase reporter constructs, corresponding miRNA mimics, and Renilla luciferase construct by Lipofectamine 2000. The cells were then lysed by 100 µl of passive lysis buffer per well on a shaker for 2 hours, and lysates were centrifuged for supernatant collection. 20 µl of lysates were then mixed with 100 µl of LAR II, and then firefly luciferase activity was measured by a single-sample luminometer. For the internal control, 100 µl of Stop & Go reagent was added to the sample. Renilla luciferase activity was then measured in the same tube. Luciferase activities between different treatments were compared after normalization with Renilla luciferase activities.

For the VerUTR competition experiments, the same luciferase system was used. In addition to 10 µg luciferase construct containing Rb1 or PTEN 3′UTR was transfected in each well, cells were also transfected with increasing folds of VerUTR plasmids and corresponding amounts of control vector to a total of 2 µg plasmid per well.

### Western Blot

Cells were seeded onto 6-well plates at 2×10^5^ cells per well overnight. They were then transfected with 1 µg of VerUTR or control vector in combination with scrambled RNA or siRNA against VerUTR. Proteins were extracted 48 hours after transfection by lysing in 60 µl of lysis buffer containing protease inhibitors (150 mM NaCl, 25 mM Tris-HCl, pH 8.0, 0.5 mM EDTA, 1% Triton X-100, 8 M Urea, and 1x protease inhibitor cocktail). Tissues were disrupted in appropriate volume of lysis buffer depending on tissue weight. All samples were subjected to SDS-PAGE and then transferred to nitrocellulose membranes followed by incubating with a rabbit monoclonal antibody against PTEN (ab32199, ABCAM) at 1∶1000 dilution, or mouse monoclonal antibody against RB1 (ab24, ABCAM) at 1∶500 dilution at 4°C overnight. The secondary antibody used was goat anti-mouse IgG at 1∶2000 dilution at room temperature for 1 hour. After detection of the protein bands, the blot was stripped and re-probed with mouse monoclonal antibody against β-actin (A5316, Sigma) to confirm equal loading. After secondary antibody incubation, the blot was washed and detected by ECL kit (Millipore) in autoradiography.

### RNA analysis

Cells (2.5×10^6^) were harvested, and total RNA was extracted with the mirVana miRNA Isolation Kit (AM1560, Ambion) according to the manufacturer's instructions. RT-PCR was performed as described recently [49], while Real-time PCR assays were performed as previously described [12], [50]. Briefly, 2 µg of total RNA was used to synthesize cDNA by reverse transcription, and the primers used are listed in SI Figure S6. miRNAs were amplified by miRNA-specific primers and poly-T primer. Comparisons between samples were made after normalization with U6 RNA levels.

### Statistical Analysis

The results (mean values ± SD) of all the experiments were subjected to statistical analysis by *t*-test. The levels of significance were set at p<0.05.

## Supporting Information

Figure S1 Colony formation affected by expression of vesicant 3′UTR. In colony formation assays, pooled cell lines were mixed in soft agarose gel and cultured in 2% FBS-containing medium. Cells transfected with control vector formed larger but less colonies than cells transfected with VerUTR.
(0.14 MB PDF)

Figure S2 Tumor sections were stained for collagen expression by applying Trichrome to the tumor sections. Keratin and muscle fibers were stained red, while collagen and bone are stained as blue or green, respectively. Cytoplasm appeared as light red or pink, and cell nuclei are dark brown to black. Within the tumor peripheral area, there were less collagen staining in the 3′UTR tumors because of slower cell proliferation and stronger cell-cell adhesion. In contrast, the area occupied by connective tissues was crowded with cancer cells in the control tumor. Scale bars, 100 um.
(0.41 MB PDF)

Figure S3 Expression of fibronectin, versican, and CD31 affected by VUTR expression. Paraffin tumor sections were stained with anti-vesicant (VCAN), fibronectin (FN), and CD31 antibodies. There was an increased staining of versican and fibronectin in the tumor comprised of cells transfected with 3′UTR and lots of small blood vessels were also identified. Control tumors showed fewer but larger blood vessels spanning at the peripheral edge. Scale bars, 100 um.
(0.29 MB PDF)

Figure S4 Conservation and targeting analysis. (a). The target sequences of miR-199a-3p and miR-144 in the Rb1 3′UTR are conserved in human and mice. Conserved nucleotides are in red and nucleotides that are complementary to miRNA are capitalized. (b). The binding site of miR-144 located within PTEN 3′UTR are conserved between human and mice. miR-136 exhibitd more than one binding sites located in the 3′UTR of PTEN and the sequences of these target sites are very conserved.
(0.01 MB PDF)

Figure S5 MicroRNAs targeting Rb1 and Pten. (a). Two additional binding sites recognized by miR-199a-3p are found in the mouse Rb1 3′UTR. miR-16, which potentially targeting Versican 3′UTR, also has a potential target site on Rb1. (b). In the 3′UTR of mouse PTEN, additional sites are found to be targeted by several Let-7 family members and miR-16, which are known miRNAs contributing to development of cancer.
(0.01 MB PDF)

Figure S6 Primers used in this study.
(0.01 MB PDF)
