# Supplementary figures and images for: Expression of Versican 3′-Untranslated Region Modulates Endogenous MicroRNA Functions
Source: PLoS One. 2010 Oct 25;5(10):e13599. doi: 10.1371/journal.pone.0013599 (PMC2963607; doi:10.1371/journal.pone.0013599)

**Vector**

**VUTR**

**Week1**

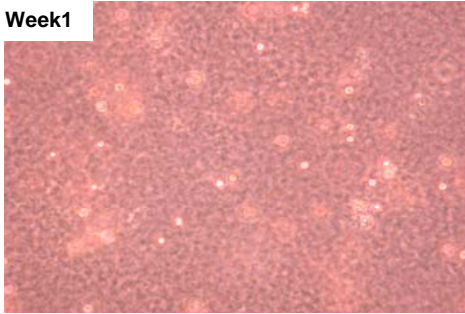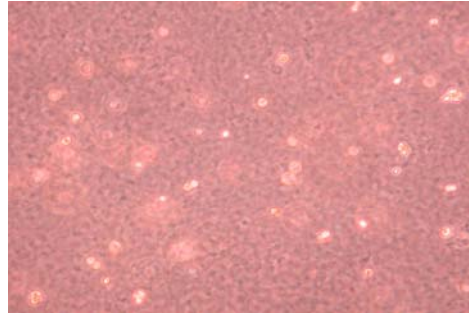

**Week2**

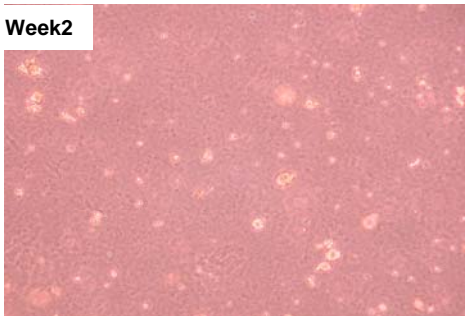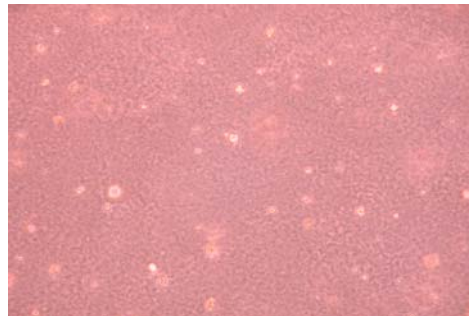

**Week3**

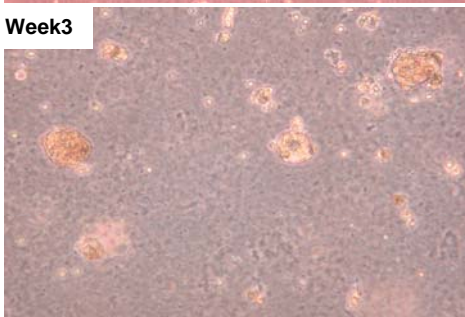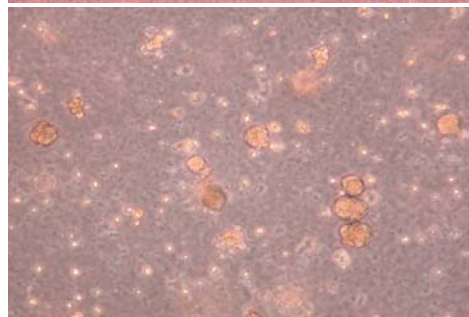

**Colony Formation**

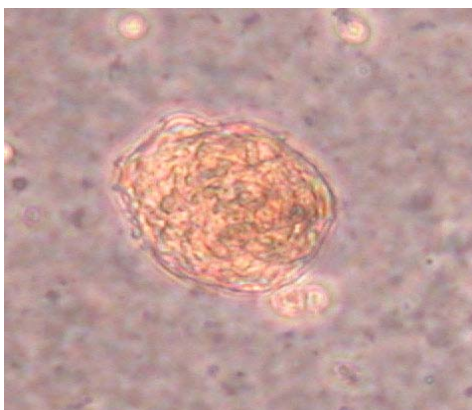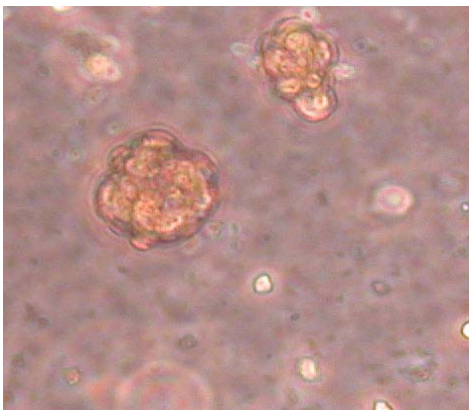

Supplement: Figure S1 — Colony formation affected by expression of vesicant 3′UTR. In colony formation assays, pooled cell lines were mixed in soft agarose gel and cultured in 2% FBS-containing medium. Cells transfected with control vector formed larger but less colonies than cells transfected with VerUTR. (0.14 MB PDF) [file pone.0013599.s001.pdf]

## Collagen staining

Vector

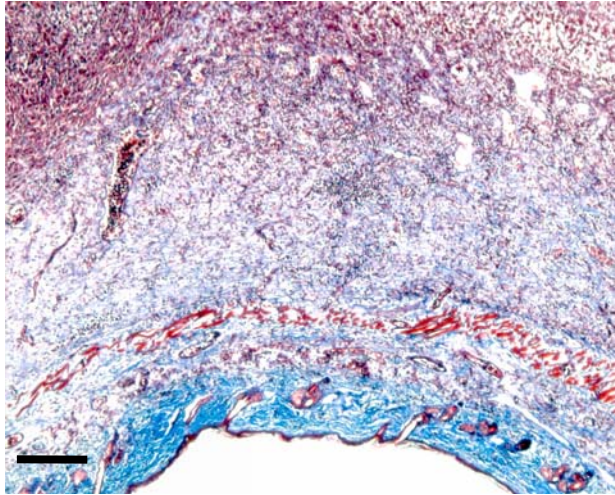

VUTR

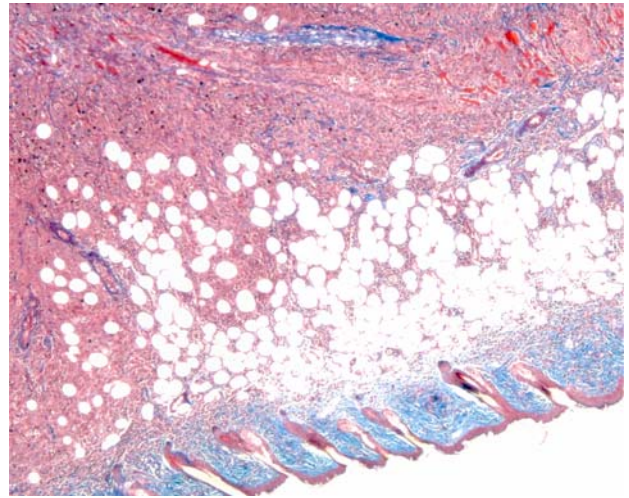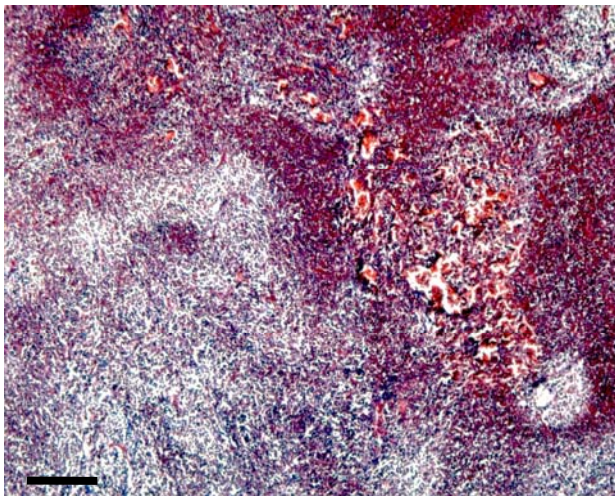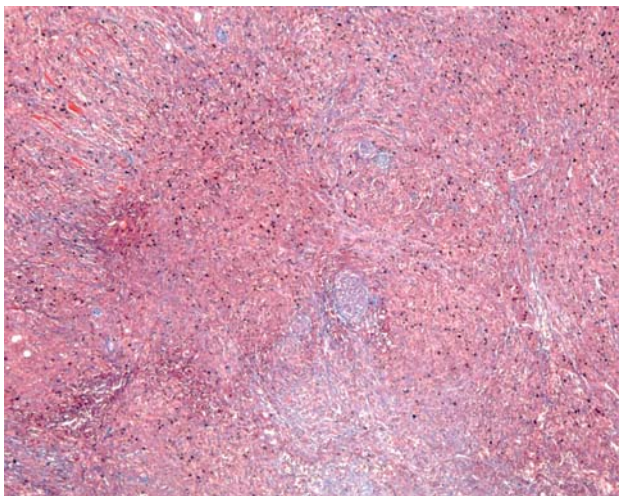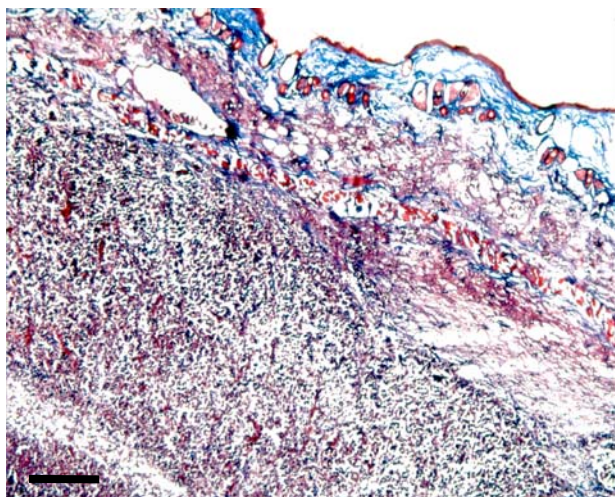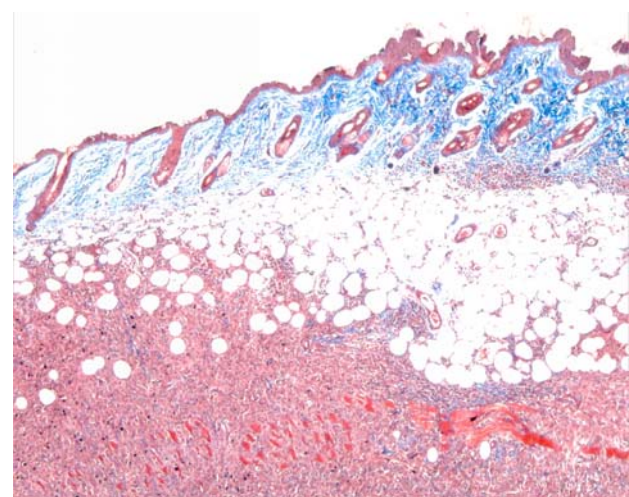

Supplement: Figure S2 — Tumor sections were stained for collagen expression by applying Trichrome to the tumor sections. Keratin and muscle fibers were stained red, while collagen and bone are stained as blue or green, respectively. Cytoplasm appeared as light red or pink, and cell nuclei are dark brown to black. Within the tumor peripheral area, there were less collagen staining in the 3′UTR tumors because of slower cell proliferation and stronger cell-cell adhesion. In contrast, the area occupied by connective tissues was crowded with cancer cells in the control tumor. Scale bars, 100 um. (0.41 MB PDF) [file pone.0013599.s002.pdf]

Vector

VUTR

FN

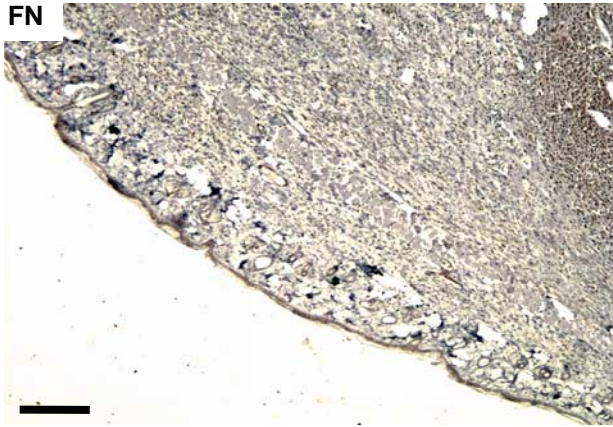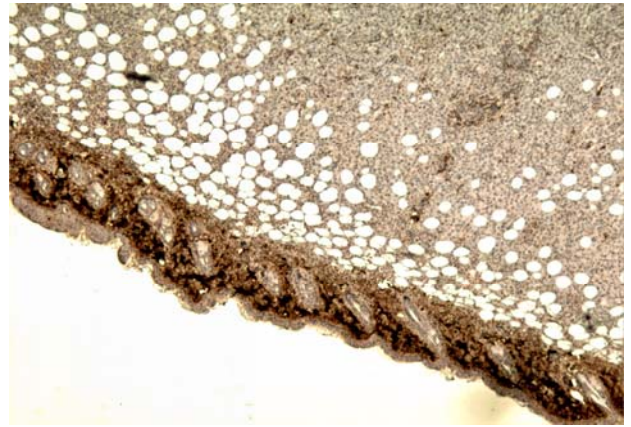

VCAN

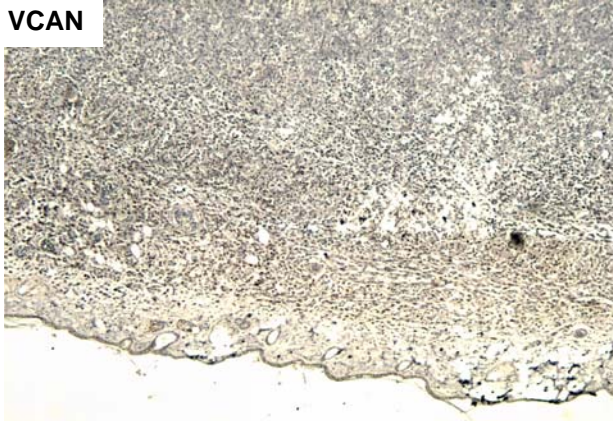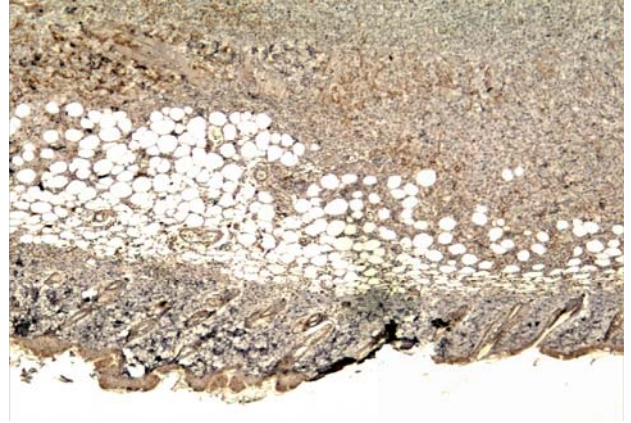

CD31

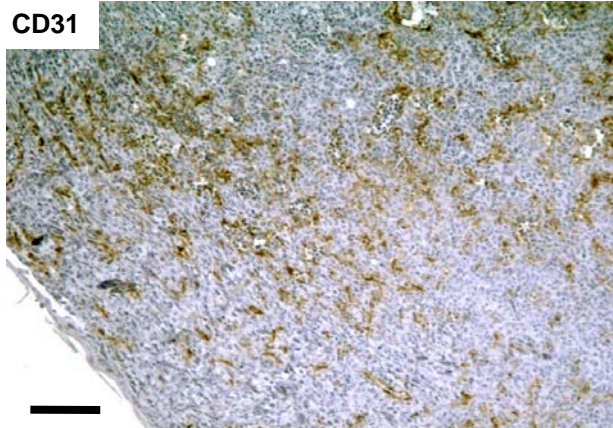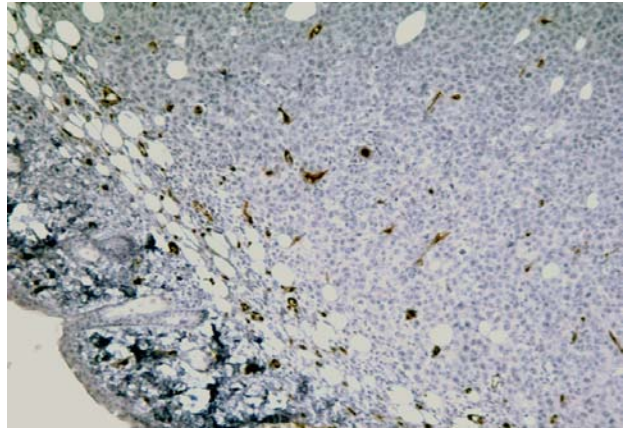

Supplement: Figure S3 — Expression of fibronectin, versican, and CD31 affected by VUTR expression. Paraffin tumor sections were stained with anti-vesicant (VCAN), fibronectin (FN), and CD31 antibodies. There was an increased staining of versican and fibronectin in the tumor comprised of cells transfected with 3′UTR and lots of small blood vessels were also identified. Control tumors showed fewer but larger blood vessels spanning at the peripheral edge. Scale bars, 100 um. (0.29 MB PDF) [file pone.0013599.s003.pdf]
