# Supplementary material for: Expression of Versican 3′-Untranslated Region Modulates Endogenous MicroRNA Functions
Source: PLoS One. 2010 Oct 25;5(10):e13599. doi: 10.1371/journal.pone.0013599 (PMC2963607; doi:10.1371/journal.pone.0013599)
Supplement: Figure S4 — Conservation and targeting analysis. (a). The target sequences of miR-199a-3p and miR-144 in the Rb1 3′UTR are conserved in human and mice. Conserved nucleotides are in red and nucleotides that are complementary to miRNA are capitalized. (b). The binding site of miR-144 located within PTEN 3′UTR are conserved between human and mice. miR-136 exhibitd more than one binding sites located in the 3′UTR of PTEN and the sequences of these target sites are very conserved. (0.01 MB PDF) [file pone.0013599.s004.pdf]

a

RB1 Conserved binding sites of miRNAs in

```
    3' auugGUUACACGUCUGAUGACa 5' hsa-miR-199a-3p
             :| | | || |||||||
654:5' augaUAcUaU-CAUACUACUGa 3' Human
614:5' augaUAcUGU-CuUACUACUGa 3' Mouse
```

```
    3' ucaugUAGUAGAUAUGACAu 5' hsa-miR-144
              |||   ||||||||
574:5' uuggaAUCugAUAUACUGUg 3' Human
603:5' uauaAUCauaUGAUACUGUc 3' Mouse
```

b

PTEN

<u>miR-144</u>

```
     3' ucAUGUAGUAGAUAUGACAu 5' hsa-miR-144
           ||| |: |  |||||||
2906:5' uuUACCUU-UAAAUACUGUu 3' Human
2907:5' uuUACCUU-UAAAUACUGUu 3' Mouse
```

<u>miR-136</u>

```
    3' agGUAGUAGUUUUGUUUACCUCa 5' hsa-miR-136
         || : |:| || ||:|||||
289:5' cCAGUUUUAUAAAAAGUGGAGa 3' Human
308:5' ccaguuUUAUAAAAAGUGGAGa 3' Mouse
```

```
    3' agguagUAGUUUUGUUUACCUCa 5' hsa-miR-136
              :|:|::: :|||||||
490:5' auucggGUUAGGG-GAAUGGAGg 3' Human
501:5'gcacuUGgguuGG-GAAUGGAGg 3' Mouse
```

```
     3' aggUAGUAGUUUUGUUUACCUCa 5' hsa-miR-136
           :|:|| ||  :||:|||||
2759:5' agGUUAUaAAcuUAAGUGGAGu 3' Human
2762:5' agGUUAUaAAcuUAAGUGGAGu 3' Mouse
```
