# Supplementary material for: Expression of Versican 3′-Untranslated Region Modulates Endogenous MicroRNA Functions
Source: PLoS One. 2010 Oct 25;5(10):e13599. doi: 10.1371/journal.pone.0013599 (PMC2963607; doi:10.1371/journal.pone.0013599)
Supplement: Figure S5 — MicroRNAs targeting Rb1 and Pten. (a). Two additional binding sites recognized by miR-199a-3p are found in the mouse Rb1 3′UTR. miR-16, which potentially targeting Versican 3′UTR, also has a potential target site on Rb1. (b). In the 3′UTR of mouse PTEN, additional sites are found to be targeted by several Let-7 family members and miR-16, which are known miRNAs contributing to development of cancer. (0.01 MB PDF) [file pone.0013599.s005.pdf]

a

Rb1

```
    3' auugGUUACACGUCUGAUGACa 5' mmu-miR-199a*
           :| ||| |  |||||||
614:5' augaUACUGU-CUUACUACUGa 3' Rb1
```

```
    3' auuGGUUACACGUCUGAUGACa 5' mmu-miR-199a*
          :::|  || ||||||:||
692:5' gcuUUGAACUGAAGACUAUUGa 3' Rb1
```

```
    3' gcGGUUAUAAAUGCACGACGau 5' mmu-miR-16
         |||| ||  :| ||||||
243:5' uuCCAACAUGAGCCUGCUGCcc 3' Rb1
```

b

PTEN

```
     3' uugauaugUUGGAUGAUGGAGu 5' mmu-let-7a
                ||: |||||:|||
3729:5' uaggcucaAAUAUACUAUCUCc 3' Pten
```

```
     3' uugauaugUUAGAUGAUGGAGu 5' mmu-let-7f
                ||| |||||:|||
3729:5' uaggcucaAAUAUACUAUCUCc 3' Pten
```

```
     3' gcGGUUAUAAAUGCACGACGAu 5' mmu-miR-16
          :||| |||  | ||:||||
4308:5' auUCAACAUU--CUUGUUGCUa 3' Pten
```
