# Supplementary material for: Expression of Versican 3′-Untranslated Region Modulates Endogenous MicroRNA Functions
Source: PLoS One. 2010 Oct 25;5(10):e13599. doi: 10.1371/journal.pone.0013599 (PMC2963607; doi:10.1371/journal.pone.0013599)
Supplement: Figure S6 — Primers used in this study. (0.01 MB PDF) [file pone.0013599.s006.pdf]

Primer	Sequence
huRB1-SacI	5’ccc ggg **gagctc** tgaggatctcaggaccttggtgg
huRB1-miR144MulI	5’ggg ccc **acgcgt** gc aaaa tttt ata aaac aag cac
huRB1-miR144MulI-mut	5’ggg ccc **acgcgt** gc aaaa tttt ata aaac aag cac **tgtca** atatc
huRB1-miR199a*MulI	5’ggg ccc **acgcgt** ca ttct gagg tat gaaa tct gttt
huRB1-miR199a*MulI-mut	5’ggg ccc **acgcgt** ca ttct gagg tat gaaa tct gttt **gtcatc** tat ga
huPTEN-SacI	5’ccc ggg **gagctc** tgaatttttttttatcaagagggat
huPTEN-miR144MulI	5’ggg ccc **acgcgt** gtg caaa gggg tagg at gtg aac
huPTEN-miR144MulI-mut	5’ggg ccc **acgcgt** gtg caaa gggg tagg at gtg aac **gtcat** tat cac
huPTEN-miR136MulI	5’ggg ccc **acgcgt** aatta ttt ccttt ct gag ca ttcc
huPTEN-miR136MulI-mut	5’ggg ccc **acgcgt** aatta ttt ccttt ct gag ca ttcc **gaggt** tt ccc
miR-199a* Realtime primer	5’ tacagtagtctgcacattggtt
miR-17-5p Realtime primer	5’caaagtgcttacagtgcag
miR-328 Realtime primer	5’ ctggccctctctgcccttccgt
Human U6 primer forward	5’ gtgctcgcttcggcagcacatatac
Human U6 primer reverse	5’ aa aaa tat gg aa cgc ttc acga atttg
mo-Gapdh1F	5’ atg gtg aag gtc tgt gtg atc atc
mo-Gapdh250R	5’ tgg gtt ctc act cct gga aga agg
